# Using global navigation satellite systems for modeling athletic performances in elite football players

**DOI:** 10.1038/s41598-022-19484-y

**Published:** 2022-09-08

**Authors:** Frank Imbach, Waleed Ragheb, Valentin Leveau, Romain Chailan, Robin Candau, Stephane Perrey

**Affiliations:** 1 Seenovate, Montpellier, 34000 France; 2 EuroMov Digital Health in Motion, Univ Montpellier, IMT Mines Ales, Montpellier, 34090 France; 3 DMeM, INRAe, Univ Montpellier, Montpellier, 34000 France

**Keywords:** Computer science, Mechanical engineering, Biomarkers

## Abstract

This study aims to predict individual Acceleration-Velocity profiles (A-V) from Global Navigation Satellite System (GNSS) measurements in real-world situations. Data were collected from professional players in the Superleague division during a 1.5 season period (2019–2021). A baseline modeling performance was provided by time-series forecasting methods and compared with two multivariate modeling approaches using ridge regularisation and long short term memory neural networks. The multivariate models considered commercial features and new features extracted from GNSS raw data as predictor variables. A control condition in which profiles were predicted from predictors of the same session outlined the predictability of A-V profiles. Multivariate models were fitted either per player or over the group of players. Predictor variables were pooled according to the mean or an exponential weighting function. As expected, the control condition provided lower error rates than other models on average (*p* = 0.001). Reference and multivariate models did not show significant differences in error rates (*p* = 0.124), regardless of the nature of predictors (commercial features or extracted from signal processing methods) or the pooling method used. In addition, models built over a larger population did not provide significantly more accurate predictions. In conclusion, GNSS features seemed to be of limited relevance for predicting individual A-V profiles. However, new signal processing features open up new perspectives in athletic performance or injury occurrence modeling, mainly if higher sampling rate tracking systems are considered.

## Introduction

Global Navigation Satellite System (GNSS) is one of the gold standard systems in position measurements in field sports. Widely used for athlete monitoring purposes^1–11^, GNSS permits discriminating the physical demand at exercise through objective mechanical parameters, computed from GNSS and Inertial Measurement Units (IMU) signals^12^. Data collected from these wearable devices provide useful insights for understanding a player’s activity and its relationship with performance outcomes or injury occurrences during practice^6,13–16^.

In most cases, the information provided by wearable GNSS devices are summarised features over a session or a period (*e.g.* distance covered at different speed intervals, averaged pace for a given interval, acceleration, and deceleration counts). Beyond a standard set of simple and easily comprehensible features, extra information of mechanical and energetic nature that is derived from the players’ position may be available for customers under the manufacturer’s policy^17–19^. However, the validity of GNSS sport receivers should be considered regarding their technical properties, such as sampling frequency. Despite a lack of accuracy for quantifying exercise demand over short distances covered at high speed, including sharp turns, GNSS suffers from error rates according to a relatively low GNSS sampling frequency^20,21^. The signal quality of receivers also depends on the spatial configuration of satellites locked for recording (*i.e.* the number of satellites and their geometrical distribution in the sky)^22^. Nonetheless, GNSS with embedded IMU stands of interest and remains prone to further technological improvements. Beyond technological aspects, practitioners mostly use summarised GNSS statistics or metrics, whereas raw data are seldom considered for player analysis. The usual data fed back from the GNSS units might be elementary, while new features extracted from raw data could be more insightful for monitoring the physical demand of exercise and the related athlete’s response.

Using GNSS data for predicting athletic performance in team sports remains challenging. First, it implies defining an athletic performance in which interactions with opponents and the environment are sufficiently lowered. Usually, assessing athletic performances requires specific testing sessions performed in controlled conditions. It comes with challenging issues due to time and investigation costs, injury exposure, psychological state disruptions, and adjustments to training plans. Nevertheless, Morin et al.^23^ recently proposed a timely method for assessing a player’s athletic performance while practicing football without performing any specific tests. In brief, the method determines individual acceleration-velocity profiles (A-V) from continuous GNSS measurements for in-game and post hoc analysis. Such profiles come with practical meanings, notably for monitoring changes in athletic properties (by analogy to force-velocity profiles). They could be used for optimising training plans or proceeding to in-game tactical adjustments in case of significant profile impairments. However, determining in situ A-V profiles for monitoring athletic performances remains at a proof-of-concept stage^23^. It should be further validated for athletic performance modeling and injury explanation purposes.

On this basis and according to the literature, the present study considers three research issues:

1. The predictability of A-V profiles using only related GNSS features
2. The value of common metrics (summarised statistics) and aggregated features that are delivered by GNSS sensors manufacturers for predictive applications
3. The use of raw GNSS data for extracting new features for prediction purposes

In order to investigate these issues, we attempted to predict A-V profiles using data from an elite football team through different modeling approaches. A baseline approach that only considers A-V profiles and dismisses any potential predictors other than historical profiles was carried out. Then, we compared it with two distinct tasks that used commercial GNSS features and features extracted from raw GNSS data.

The rest of the manuscript is organized as follows. We introduce the data set that highlights the predictor and outcome variables. Next, we introduce the proposed models and their variants. Accordingly, we present the obtained results followed by exhaustive discussions of these results before concluding our study in the last section.

## Methods

This section introduced a descriptive analysis of the data set used in our experiments. We defined the predictors and outcome variables besides the considered problem formulations before elaborating the proposed models. For clarification, we provided a pseudo-code of the modeling methodology through Algorithms 1, 2 and 3.

### Data set

#### Population studied

Data from the *FC Lucerne* football club were collected over a 1.5 season period (2019–2021). The team evolves in the Superleague division, the highest division in Swiss professional football. A total of 196 training sessions and 74 games were stored in a cloud-hosted multi-model database (ArangoDB, CA, USA). For each session, raw GNSS data (Fieldwiz V1, CH, with concurrent reception of Global positioning system, Galileo, GLONASS, and BeiDou systems) and summarised features (see Appendix A for details) of each player were stored in a database as json files. A total of 42 players were initially recorded, including regular professional players and young hopes. Participants were fully informed about data collection, and their written consent were obtained. The study was performed in agreement with the standards set by the declaration of Helsinki (2013) involving human subjects. The protocol was reviewed and approved by the local research Ethics Committee (EuroMov, University of Montpellier, France). The present retrospective study relied on the collected data without causing any changes in the training plans of football players.

#### Predictor variables

Predictors are summarised in Appendix 1, Table A1. Let $\mathcal {X} \subset \mathbb {R}^d$ with $d \in \mathbb {N}$ be the domain of definition of the random variable $X = \{x_1, \ldots , x_d\}$. The variable *X* is thus defined as a vector of *d* dimensions, composed of aggregations of the summarised features given by the GNSS software (Fieldwiz, ASI, CH). Aggregated features can take two forms:

1. The average of summarised features $\bar{X}= \{\bar{x_1}, \ldots , \bar{x_d}\}$, such that:
  1 $$\begin{aligned} \bar{X}_{i} = \frac{1}{N} \sum _{j=1}^{N} X_{i,j} \, \quad , 1 \le i \le d \end{aligned}$$
2. An exponential weight according to a *softmax* function (see Eq. 2), such that:
  2 $$\begin{aligned} X_d = \sum _j w_j X_{d, j} \quad \text {where} \quad w_j = \sigma (\varvec{t})_j^{\beta } = \frac{e^{-\beta t_j}}{\sum _{k=1}^{K} e^{- \beta t_j}} \quad \forall j \in \{1, \ldots , K \} \, . \end{aligned}$$

In Eq. 2, $X_d$ denotes an aggregated feature weighted by a scaling factor $w_j$. $w_j$ is determined by a *softmax* function $\sigma (\varvec{t})_j^{\beta }$ in which $\varvec{t}$ is a time vector describing the distance of events to the game of interest and $\beta$ denotes a scale parameter that sets the sensibility of the exponential decay weighting function.

For both aggregation methods, we arbitrarily set a window *L* of size $L = 5$. It refers to the summarised predictor sets given by the GNSS software that are pooled according to the last *L* sessions (either training or game) preceding the game of interest. Since the frequencies of sessions are heterogeneous, the number of days preceding the game to be predicted may differ over the weeks.

#### Outcome variables

In order to investigate the effect of training on athletic performances, we relied on A-V profiles such as provided by^23^ but in a slightly different way. Individual A-V profiles were modeled for each games. From the raw velocity $\mathbf {V}$ and a sampling frequency $\omega$, we derived an acceleration *A* such that

$$\begin{aligned} A_i(nT) = V_i(nT) - V_{i-1}(nT) \, , \quad T=1/\omega \quad \text {and} \quad \omega = 10Hz \, . \end{aligned}$$

Here, we consider a signal $x(t) \rightarrow x \left[ n \right]$ with $x \left[ n \right] = x (nT)$ being the discrete formulation of *x*(*t*).

**Figure 1:**
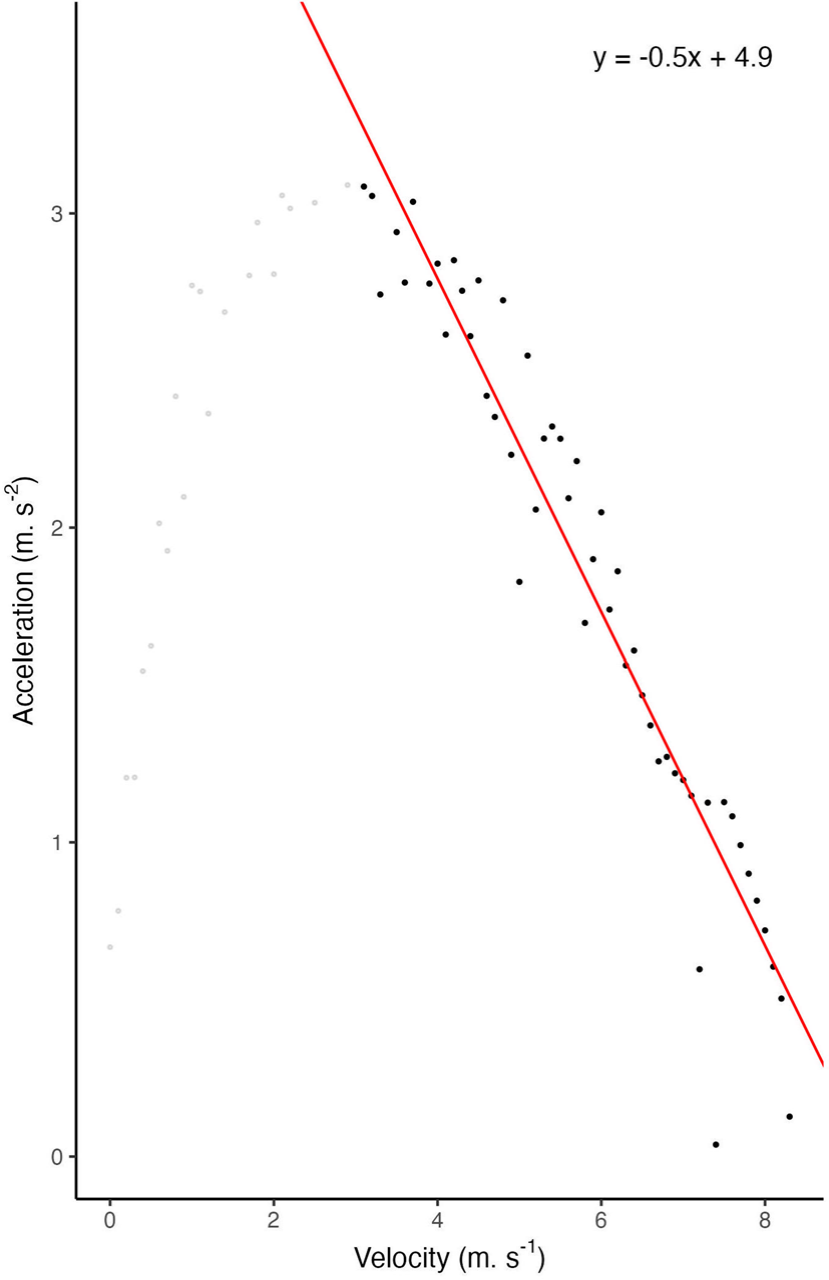
Example of A-V profile modeled for a given player and a randomly selected game. Only plain dots (velocities above 3 m s^−1^) were used for fitting the linear regression.

Then, a first-order Butterworth filter was applied to the acceleration signal with a cut-off frequency of 1 Hz. Velocity observations were binned into $0.1 \, \text {m}.\text {s}^{-1}$ width bins in which the maximal acceleration values were retained. Hence, we modeled A-V profiles over velocities superior to $3 \, \text {m}.\text {s}^{-1}$ using a linear regression between acceleration and velocity (see Fig. 1). A total of 1032 profiles were modeled, for an average of $25.80 \pm 20.37$ per player. The large standard deviation is related to occasional players (*e.g.* young players) who only played a few games through the season.

**Figure 2:**
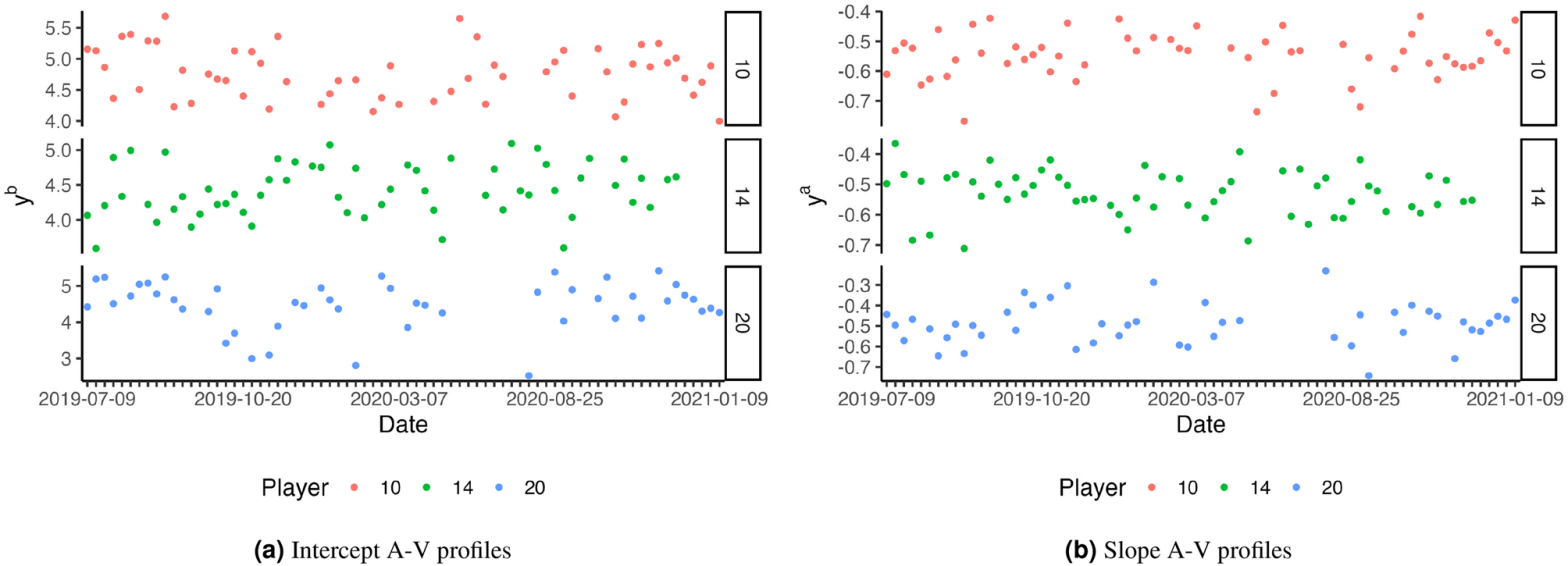
Evolution of A-V profiles fitted intercept and slopes over the 1.5 season period. Three players are randomly selected.

The performance definition is given by $\big \{ Y^a, Y^b \big \} \in \mathcal {Y}$ such that $Y^a$ and $Y^b$ refer to the corresponding slope and the intercept of individual A-V profiles, respectively. Therefore, each observation in the ensemble $\big \{ y^{a}_{j,t}, y^{b}_{j,t} \big \} \in \big \{ Y^{a}, Y^{b} \big \}$ is related to both an athlete *j* and the day of realisation *t*. A sample of fitted coefficients is presented in Figure 2. To formalize, letting $X \times Y \sim f$ with a density function *f*, the built data set is a sample $S = \Bigg \{ \left( x_j, y^{p}_{j} \right) \Bigg \}_{j \le n} \sim f^{n}$ .

A descriptive analysis of A-V coefficients reported mean values and standard deviation as a noise estimate. Accordingly, we have $\mu _a = -0.533 \pm 0.078$ and $\mu _b = 4.721 \pm 0.483$ for $Y^a$ and $Y^b$, respectively.

### Definition of models and multivariate approaches

#### Time-series forecasting

Time-series forecasting problems are dominant in sports, applied to game outcomes^24–27^ generally intended to tipsters or bookmakers, sports popularity^28^, or performance monitoring^29^. In our study, we first consider that the auto-regressive component of the target variable may be influential in the prediction of individual athletic performances. Therefore, we started the modeling by defining baseline prediction performances from time-series forecasting, only using games observations (excluding training sessions).

In time-series forecasting, models without covariates use a restricted data set in which predictors are merely time information. The forecasting is deduced from the information in trend and seasonality components. In order to find the most performing models for time-series forecasting, we proceeded with a model selection using a simple holdout procedure, according to a split ratio of 0.8 between the training and testing data set. We can righteously expect a linear relationship between changes in theoretical maximal acceleration and maximal running velocity. Consequently, the ensemble $\{ Y_a, Y_b \}$ was predicted in two different ways: sequentially (uni-modal forecasting) and concurrently (multi-modal forecasting).

Afterwards, we benefited from the selected forecasting models by combining them into a weighted average ensemble for better performances than a randomly selected single model on average.

#### Ridge regularisation

Then, we addressed the problem of predicting the acceleration-velocity profile from GNSS summarised features. Changes in A-V fitted parameters were investigated using a predictive regression approach. For this, a linear model (a ridge regularisation) used features pooled according to the two aforementioned aggregation methods (see Eqs. 1 and 2) and were compared to a Long Short Term Memory (LSTM) neural network, a particular case of recurrent neural networks (RNN).

Using a ridge regularisation was motivated by the high dimensional context that may lead to unsteady multivariate linear models, excessively sensitive to an expanded space of solutions. Accordingly, ridge regularisation reduces the space of solutions while solving collinearity problems, which remains common in sports^30–32^. It thus prevents biased estimates through penalising estimates of correlated features^33,34^. According to the two aggregation methods, the multivariate linear models $m^{ridge} : X_p \rightarrow Y$ and $m^{ridge^{*}} : X_{p^{*}} \rightarrow Y$ take the general formulation

3 $$ y_{t}^{{({\text{ridge}})}}  = {\mathbf{x}}_{{\mathbf{t}}} ^{t} \beta  + \varepsilon _{t} , $$

where $\varvec{x} \in X_{p}$ denotes the pooled predictors according to the *mean* function (see Eq. 1) and $\varvec{x} \in X_{p^{*}}$ refers to the pooled predictors according to the exponential weighting function (see Eq. 2) for $m^{ridge}$ and $m^{ridge^{*}}$, respectively. Also, $\beta \in \mathbb R^{d}$ denotes the parameters of the model and $\epsilon _t$ the random error term.

In addition, we defined a *control* task in which we attempted to predict $\big \{ y^a_{j,t}, Y^b_{j,t} \big \}$ from $\varvec{X}_{j,t}$. Using commercial features of the day of A-V profile realisation should be, in theory, the simplest regression task and provide the lowest error rates in prediction.

#### Long short term memory neural network

**Figure 3:**
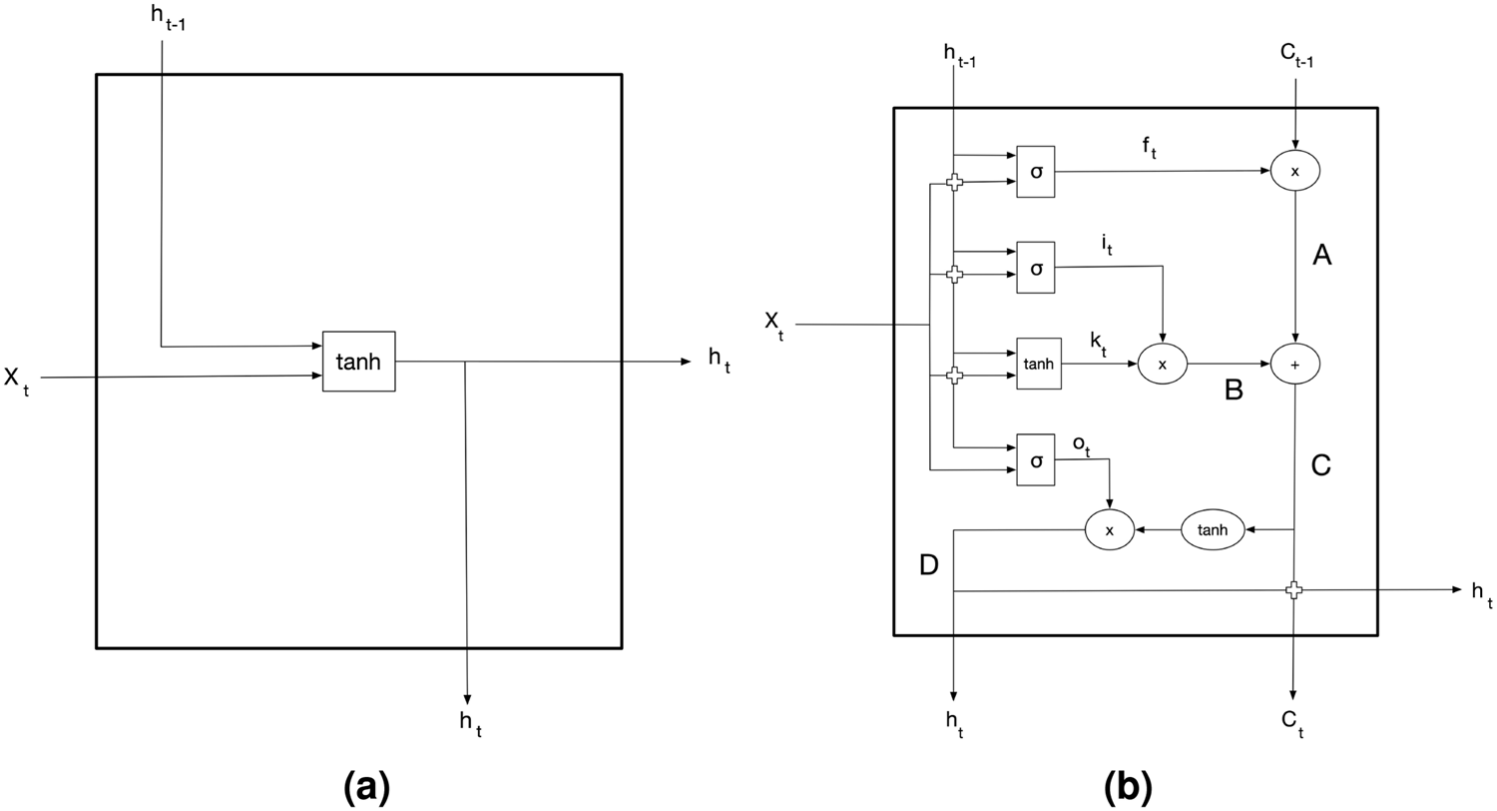
Simplified diagram of (**a**) a RNN cell and (**b**) a LSTM cell.

Recurrent neural network is the class of neural networks that considers past information to be used as inputs while preserving the hidden states. Let us consider a multidimensional vector $\varvec{X}$ of fixed length *l* and dimension *d*, which includes unpooled summarised features as the model’s input. Basically and from a $l \times d$ matrix, a recurrent unit successively combines the current values of $\varvec{X}_t$ of size *d* with the predicted value at time $t-1$ to return an output $\varvec{h}_t$, defined by a function $f(\varvec{X}_t, \varvec{h}_{t-1})$ (see Fig. 3a). This procedure is repeated as many times as the number of training sessions preceding a game in a multi-layered structure. However, RNN suffers from short-term memory due to a vanishing gradient problem. Nevertheless, used for updating neural network weights, a gradient that shrinks as it back propagates through time stops the learning of layers. These layers may thus cause a loss of past information, particularly with long sequences.

Introduced by Hochreiter et al.^35^, LSTM neural networks are designed to conserve long-term information through extended internal mechanisms. LSTM architecture benefits from a cell state and various gates that regulate the flow of information. As shown in Fig. 3b the cell state maps the previous cell state $\varvec{C}_{t-1}$ to a new cell state $\varvec{C}_{t}$ in which all the relevant information is carried throughout the sequence and where gates add or remove information to/from it. More details about LSTM dynamics in handling recurrent sequences are available in the original reference^35^. In sports, the use of LSTM remains quite recent with applications among action and activity recognition^36–40^, game outcomes^41^ and sports related concussion^42^.

### Multivariate modeling approaches

#### Using commercial features

In order to investigate the effect of training sessions on changes in athletic performances, a multivariate analysis that includes data from training sessions is required.

We aimed at predicting $\{ Y_a, Y_b \}$ using two sets of aggregated predictors $X_p$ and $X_{p^{*}}$ from the original features displayed in Appendix A, Table A1. Since models rely on several predictors, we consider the multivariate modeling approach.

For multivariate models, we performed a feature selection based on F-statistics and p-values converted from the cross-correlation between each feature of interest and the target through univariate sequential linear regression tests. Accordingly, we held the ten most meaningful features for making further predictions.

#### Extracting new features from raw global navigation satellite system data

In the previous formulation, the player position was recorded by GNSS at a sampling frequency of 10 Hz. Timestamp, player position (*i.e.* latitude, longitude), and velocity were available. Since commercial features were computed from the raw velocity vector $\mathbf {V}$ and its derivative $\mathbf {A}$, we proposed to extract new features directly from $\mathbf {V}$.

First, we consider $\mathbf {V}$ being a stationary time-series $(X_t)_{t \in \mathbb {R}}$. Formally, a time series is stationary if the law $\mathcal {L}$ of a generated vector $(X_{t_{1}}, \ldots , {t_{n}})$ is time translation invariant. That is, we consider a law $\mathcal {L}$ such as $\mathcal {L} (X_{t_{1}}, \ldots , {t_{n}}) = \mathcal {L} (X_{t_{1}+h}, \ldots , X_{t_{n}+h}), \quad \forall (t_1, \ldots , t_n) \in \mathbb {R}^n \quad and \quad h \in \mathbb {R}$ with *t* being a time value and $\mathbb {R}$ being a set of real numbers^43^. The stationary of time-series was checked using a Dickey-Fuller test^44^.

Several features were extracted from the time series in both time and frequency domains through Discrete Fourier Transform. For this purpose, we used the *tsfresh* Python module^45^. The feature extraction from both domains provided categorized 779 features^46^. A feature selection like performed during the previous tasks let us retain only the ten most relevant features, according to their significance level (*F* statistic and *p* value).

In summary, pseudo-code of the algorithms used in the methods is provided in Appendix 1, Section A.3.

### Statistical analysis

In prediction, model performances were characterised by the mean absolute percentage error (%, MAPE) computed on test data sets. Repeated measures analysis of variance (ANOVA) and post-hoc analysis highlighted the significance of differences in MAPE distribution between models. Depending on a reference model for comparison, Tukey’s or Dunnett’s *p* value adjustment was used. The marginal mean difference $\beta _{diff}$ was reported along with 95 % confidence intervals. Partial $\eta ^{2}$ (or $\eta ^{2}$ for one-way ANOVA) values were reported as a measure of effect size in ANOVAs. The significance level was set at *p* = 0.05 and consistently reported within the analysis.

## Results

### Predicting A-V profiles from games: reference models

The first baseline prediction was described by error rates observed in the *control* task. Using a set of predictors to predict A-V coefficients of the same session using a ridge regularisation returned an average MAPE of 0.066% and 0.102% for intercept and slope, respectively.

**Figure 4:**
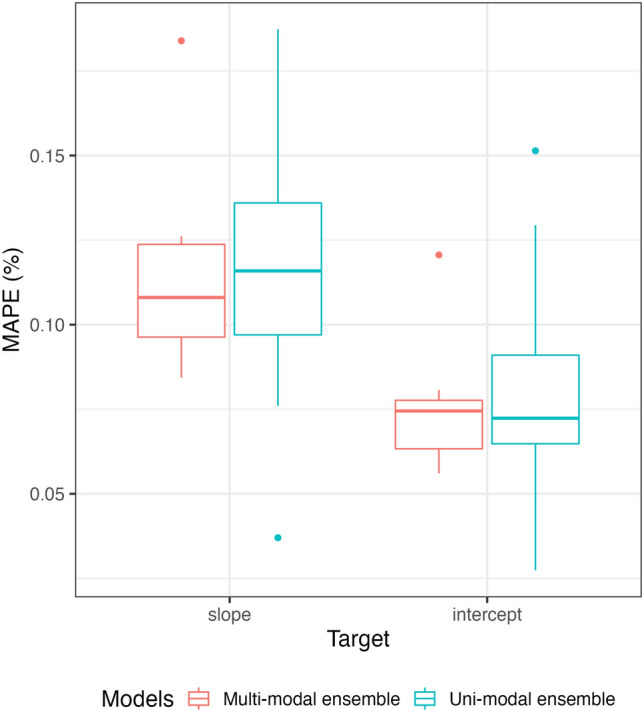
Distributions of MAPE regarding multi-modal and uni-modal ensemble forecasting models.

As shown in Fig. 4, we observed likely different MAPE values between intercept and slope predictions of A-V profiles. For this reason, we considered linearly re-scaled coefficients due to range and variance differences (averaged range = 0.325 and range = 3.98; $\sigma ^{2} = 0.006$ and $\sigma ^{2} = 0.246$ for the slope and the intercept, respectively). Accordingly, a two-way repeated measure ANOVA showed a slight trend in favour of an easier prediction task on A-V intercept ($\beta _{diff} = -0.011 \, \in \, [-0.07, 0.003] \, 95 \, \% \, CI, p = 0.122$).

**Figure 5:**
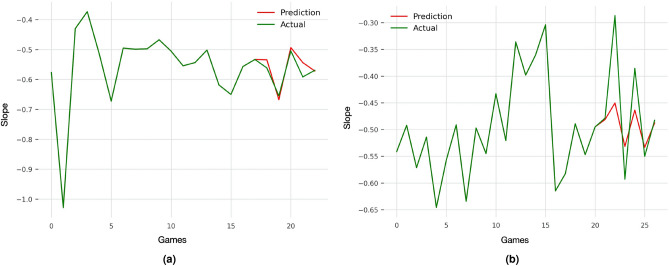
Example A-V profiles slopes forecasting using the uni-modal averaged ensemble. (a) represents the best prediction, (b) is the median prediction. Note that the red line represents the prediction made on the testing data set.

Average ensembles were built following a model selection of a large set of time-series forecasting models $\mathcal {M}_{ts}$. In the uni-modal approach, the forecasting models which provided the lowest MAPE in prediction were Prophet^47^, Theta, FourTheta^48^, and Fast Fourier Transform based. As expected, the combination of these models into an averaged ensemble provided the best performances (see Fig. 5 for examples). In the multi-modal approach, the retained forecasting models were VARIMA^49^, RNN-LSTM, and auto-regressive encoder-decoder Transformer^50^. In this case, RNN-LSTM and the averaged ensemble provided the best performances for predicting A-V slopes and intercepts, respectively. However, multi-modal averaged ensembles provided only a slight trend for a greater accuracy and were not significantly more accurate than univariate ensembles models on average ($\beta _{diff} = -0.004 \, \in \, [-0.012, 0.020] \, 95 \, \% \, CI, p = 0.541$). Overall synthesis of the selected forecasting models and their performances are presented in Table 1.

In comparison to the simplest scenario in which A-V profiles coefficients are predicted from a set of commercial features from the day of A-V realisation ($\big \{ y^a_{j,t}, Y^b_{j,t} \big \}$, the *control* task), averaged ensembles forecasting models tended to be less accurate ($\beta _{diff} = 0.013 \, \in \, [-0.002, 0.028] \, 95 \, \% \, CI, p = 0.095$).

**Table 1:** Average MAPE for each selected model.

Models	MAPE_slope_	MAPE_intercept_	Multi-modality^b^
Prophet	0.134	0.095	×
Theta	0.150^a^	0.096	×
FourTheta	0.120	0.085	×
FFT	0.161	0.121	×
Ensemble	**0.115**	**0.081**	×
VARIMA	0.162	0.127	✓
RNN-LSTM	**0.111**	0.099	✓
Transformers	0.120	0.075	✓
Ensemble	0.113	**0.072**	✓

Significant values are in [bold].

^a^Additive seasonality.

^b^Multi-modal models required longer time-series. We limit the study of these models to time-series larger than 40 observations.

Time series forecasting models are considered as a reference for further performance predictions and model comparisons.

### A multivariate modeling using data from past training sessions and games

Analysis of re-scaled MAPE a lower error rate when predicting the intercept coefficients ($\beta _{diff} = -0.013 \, \in \, [-0.021, -0.005] \, 95 \, \% \, CI, p = 0.002, \, \text {partial} \, \eta ^{2} = 0.06 \, \in \, [0.01, 0.15] \, 95 \, \% \, CI$). Post-hoc comparisons showed that multivariate LSTM and ridge regression that used data from past training and games sessions provided a higher error rate than the ridge regression of the *control* scenario. However, no significant differences in MAPE were observed between multivariate time-series forecasting models and regularised regression (LSTM and ridge regularisation). On average, individually fitted models did not provide lower prediction errors than those fitted on the group (*p* = 0.381). Except for univariate time-series forecasting models which only considered data from games and multivariate LSTM, there was no advantage of using the exponentially weighted aggregation (refer to Eq. 2 for details) over a simple aggregation according to the mean ($\beta _{diff} = 0.001 \, \in \, [-0.010, 0.012] \, 95 \, \% \, CI, p = 0.844$).

No significant difference was reported between averaged intercept and slope predictions in models that used features extracted from raw data. Only a slight trend for a lower MAPE was imputed to intercept predictions ($\beta _{diff} = -0.012 \, \in \, [-0.027, 0.002] \, 95 \, \% \, CI, p = 0.09$). In addition, individual and group computed LSTM provided similar performances in terms of accuracy (*p* = 0.775). That discarded any advantage of building models per player for predictions.

**Figure 6:**
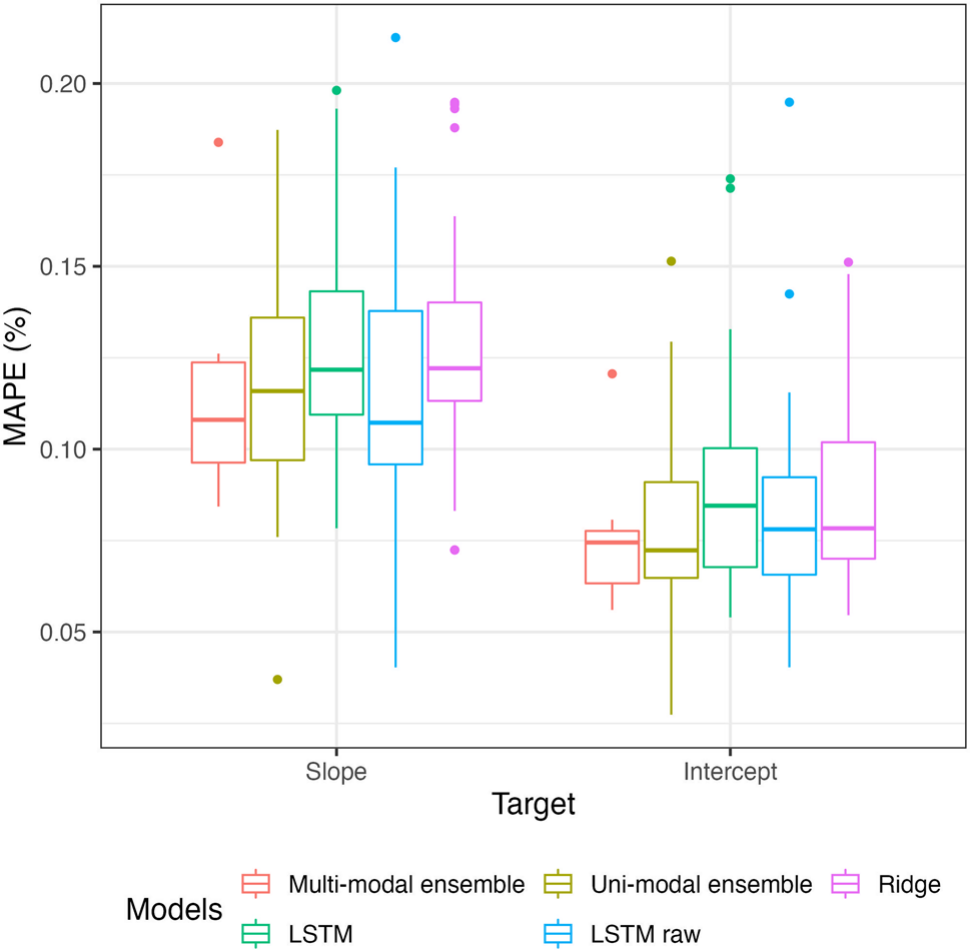
Distributions of models’ MAPE.

An overview of model performances showed that in average, the *control* task (the prediction of A-V profiles from commercial features of the same game) provided a lower error rate than any modeling task using past data ($\beta _{diff} = -0.019 \, \in \, [-0.031, -0.008] \, 95 \, \% \, CI, p = 0.001, \, \eta ^{2} = 0.03 \, \in \, [0.01, 0.08] \, 95 \, \% \, CI$). In addition, neither models that used commercial features, nor models that considered new features extracted from raw data outperformed the time-series forecasting ensembles (*p* = 0.124, see Fig. 6). No significant differences in error rate distribution were found between the source of features (*p* = 0.453).

**Table 2:** Summary of models performances according to intercept and slope coefficients.

Model	Target	Population	Aggregation	$\overline{MAPE}$
**Multi-modal Ensemble**	Intercept	I	N/A	**0.076**
LSTM (raw)	Intercept	G	N/A	0.077
Ridge	Intercept	G	Exponential	0.080
Ridge	Intercept	G	Mean	0.080
Uni-modal Ensemble	Intercept	I	N/A	0.080
LSTM	Intercept	G	Exponential	0.084
LSTM	Intercept	G	Mean	0.084
Ridge	Intercept	I	Mean	0.085
Ridge	Intercept	I	Exponential	0.085
LSTM (raw)	Intercept	I	N/A	0.088
LSTM	Intercept	I	Mean	0.089
LSTM	Intercept	I	Exponential	0.090
**LSTM**	Slope	G	Mean	**0.114**
LSTM	Slope	G	Exponential	0.114
Uni-modal Ensemble	Slope	I	N/A	0.115
Multi-modal Ensemble	Slope	I	N/A	0.116
RIDGE	Slope	G	Mean	0.116
RIDGE	Slope	G	Exponential	0.116
LSTM (raw)	Slope	G	N/A	0.119
LSTM (raw)	Slope	I	N/A	0.121
LSTM	Slope	I	Mean	0.126
RIDGE	Slope	I	Mean	0.128
LSTM	Slope	I	Exponential	0.128
RIDGE	Slope	I	Exponential	0.129

$\overline{MAPE}$ represents the averaged MAPE over individuals and validation folders. The population represents either models computed over the group of players (G) or individually computed models (I).

Significant values are in [bold].

## Discussion

This study compared two multivariate modeling approaches that use commercial features and features extracted from GNSS raw data to a time-series forecasting approach. We considered the last as *reference* models that account for past events for predicting the A-V profiles of the next game. Beforehand, predictions made using predictors of the game of A-V profile realisation (*i.e. control* models) informed their predictability in ecological conditions.

Concerning the reference models, performing multi-modal forecasting might provide better results, but it also requires a larger sample size than uni-modal forecasting methods to estimate model parameters correctly. Accordingly, we filtered out players who performed less than 40 games for computing multi-modal forecasting models. Only nine players (out of 42) were retained for prediction, whereas the uni-modal task included data from a larger population (19 players). Therefore, the sample size heterogeneity should be considered when interpreting the forecasting results since a larger sample size might reasonably provide different, if not better, forecasting performances.

A practical limitation of univariate forecasting models is that we only consider game data for prediction. Hence, interpretations drawn from each forecast are restricted to the effect of preceding games on the next game, and the contribution of training sessions preceding a performance remains unclear. Accordingly, technical and medical staff around players should exploit multivariate models for detecting key performance indicators (KPIs) of A-V changes, or any other outcome^51^.

Being based on commercial summarised statistics (*i.e.* returned by the manufacturer, see Appendix 1, Table A1) or features extracted from the velocity vector, it was likely easier for the model to predict A-V profiles’ intercepts than the slopes. A greater variance allocated to this parameter may reasonably explain that finding, easing the estimation of the coefficient regarding a random error. In practice, a small change in the A-V profile may result in a substantial modification of the theoretical normalised force output (*i.e.* the acceleration) at the onset of maximal locomotor activities.

When comparing multivariate to *reference* forecasting models (uni-modal and multi-modal), no significant differences in error rates suggest that features describing past sessions were not informative enough to improve predictions. Accordingly, time stands as a significant predictor variable of subsequent events.

In addition, ridge regularisation used pooled features according to a simple aggregation by the mean or exponential smoothing. However, changing one pooling method to another did not lower prediction error rates. At first glance, that indicates either a limited relevance of the explanatory features used in the model or a lack of A-V profile predictability. However, the low error rates of predictions provided by *control* models allow us to support a reasonable A-V profile predictability.

In a small sample context, using a larger population may lead to more robust estimates of parameter coefficients. One possible way could be to build models over a group of players instead of a model per player^32^. Our results did not confirm such benefits since there is no benefit to using player-specific models for predicting A-V profiles with the current data.

An overall analysis and model comparison highlight that despite slight differences between top and bottom model ranking (see Table 2), no significant differences in prediction errors were reported. Accordingly, neither commercial, new features extracted from time and frequency domain analysis nor the pooling methods and model framework (time-series forecasting or multivariate regressions) led to significantly better prediction of A-V parameters. Once again, it questions the relevancy of GNSS-based features for modeling physiological adaptations to training^52^ or their value for explaining outcomes under a substantial opponent influence. It is essential to point out the lack of information for the GNSS signal quality. As mentioned in the introduction, GNSS signal accuracy relies on time/frequency and spatial parameters. The receiver manufacturer used in our study (Fieldwiz V1, CH) did not store any spatial accuracy factor such as horizontal dilution of precision. Therefore, we recommend the manufacturer to report signal quality details for practical use and research purposes^53,54^. Nevertheless, using features not based on expert hypotheses but fully extracted from signal processing methods appeared to be as valuable as the commercial ones. It leverages information that could be drawn from GNSS data and opens the way to future works on data mining and knowledge discovery in the sports field. However, this perspective comes with feature interpretability issues, particularly those related to the frequency domain.

Features retained for regression after the feature selection procedure reveal KPIs of A-V profile changes. Based on a top ten representation (see Appendix 1, Table A2), we could state that the distance covered at high intensity is not necessarily the highest value when regarding other variables, such as the number of accelerations and decelerations for specific intensity bands. Such KPIs should help guide field and resistance training regarding individual objectives. However, since multivariate models suffered from explanatory power regarding *reference* models (*i.e.* univariate forecasting models), interpretation of the selected features for practical application should be made with caution.

Finally, when considering re-scaled MAPE, prediction errors varied between 7% and 10%. We believe this is an acceptable accuracy since the A-V profile depends on unmeasured and uncontrolled factors, namely the opponent activity, then any psychological, environmental, or nutritional aspects. Therefore, GNSS wearable sensors could stand of value, though limited, for prediction purposes and more generally included in athlete monitoring systems while estimating external training loads^9^. Regardless, in light of the above limits, monitoring processes should be carried out under a more data-informed than the data-driven approach in which external training indicators are monitored along with internal markers, and environmental factors^55^.

In our study, we provided a simple estimation of the A-V predictability through the *control* task, which benefits from the relationship between the commercial features and the modelled profiles of a given game. However, a deeper analysis of the A-V estimator noise and heteroscedasticity of the outcome variables should be carried out in a future study.

Beyond predictive applications, A-V profiles provide relevant insights regarding the theoretical maximal isometric force of hip extensors (*i.e.* through the profile intercept) and the capacity to produce a significant level of horizontal force at high velocities (*i.e.* according to the slope of A-V relationship). These mechanical factors may be key determinants of soft-tissue injury occurrence^56^, short sprint performances^57^ and could guide individual training prescription.

The technological rise provides higher sampling frequency systems (*e.g.* IMU and motion capture systems) as compared to GNSS devices, intended for discriminating exercise and its demand in ecological conditions. A physiological representation of the responses to exercise may be therefore extracted. Besides, going through raw data recorded by these systems may contribute to solving the enigma of injury occurrence, which remains a hot research area in sports science with major economic repercussions^58,59^.

## Conclusion

In this study, we aimed at modeling coefficients of individual A-V profiles. For this purpose, we first considered time-series forecasting models, which used data from games only as the baseline of models’ performances. Then, multivariate modeling approaches were compared to these baseline models with a regression task using a regularised linear regression (ridge) and a neural network architecture (RNN-LSTM). Two distinct functions were employed to aggregate training sessions predictors; a *mean* and an exponential weighting function (both of them are defined in Eqs. 1 and 2). Finally, we extracted new signal processing features from the GNSS raw data and assessed their contribution to the modeling process. We recall that except for time-series forecasting, models were fitted either per player or over the group of players. Overall, no method showed significant better performances in prediction than the time-series forecasting. Global navigation satellite system features seemed to be of limited relevance for predicting individual in-situ A-V profiles. However, time-domain and frequency-domain features extracted from the raw data outlined the potential of signal processing methods for extracting new information. That opens up new perspectives in athletic performance or injury occurrence modeling, using IMU and movement tracking systems concurrently.

## Key points

- Global navigation satellite systems are valuable for modeling in-situ A-V profiles. However, its predictability using GNSS-derived features from training sessions remains limited.
- Multivariate modeling highlights key performance indicators of A-V changes among commercial, training-related features. Alternatively, signal processing methods pave the way to new modeling perspectives of performance and injury modeling, mainly if applied to measurement systems with higher sampling rates (*e.g.* IMUs).
- A-V time-derived features are likely as relevant as GNSS-based features for explaining changes in A-V profiles. It emphasizes the necessity for multidimensional modeling while considering the opponent’s activity, psychological and environmental factors.

## Supplementary Information

Supplementary Information.

## Data Availability

The data sets generated during and/or analysed during the current study are not publicly available due to property of FC Lucerne but are available from the corresponding author on reasonable request.
