# Supplementary material for: Using global navigation satellite systems for modeling athletic performances in elite football players
Source: Sci Rep. 2022 Sep 8;12:15229. doi: 10.1038/s41598-022-19484-y (PMC9458673; doi:10.1038/s41598-022-19484-y)
Supplement: Supplementary file 1 — Supplementary Information. [file 41598_2022_19484_MOESM1_ESM.pdf]

Using Global Navigation Satellite Systems for modeling Athletic Performances in elite football players

Frank Imbach[1,2,3,*], Waleed Ragheb[1,+], Valentin Leveau[1,+], Romain Chailan[1], Robin Candau[3], and Stephane Perrey[2]

[1]Seenovate, Montpellier, 34000, France
[2]EuroMov Digital Health in Motion, Univ Montpellier, IMT Mines Ales, Montpellier, 34090, France
[3]DMeM, Univ Montpellier, INRAe, Montpellier, 34000, France
[*]frank.imbach@umontpellier.fr
[+]these authors contributed equally to this work

A Appendix 1

A.1 Data set description

Table A1. Summary of explanatory variables computed by the manufacturer

Predictor variables	X_i	Description
TD	X_1	Total distance covered by a player (km)
D_{0-5}	X_2	Distance covered at a running speed below $5\,km.h^{-1}$
D_{5-10}	X_3	Distance covered at a running speed between $5\,km.h^{-1}$ and $10\,km.h^{-1}$
D_{10-15}	X_4	Distance covered at a running speed between $10\,km.h^{-1}$ and $15\,km.h^{-1}$
D_{15-21}	X_5	Distance covered at a running speed between $15\,km.h^{-1}$ and $21\,km.h^{-1}$
D_{21-24}	X_6	Distance covered at a running speed between $21\,km.h^{-1}$ and $24\,km.h^{-1}$
D_{24-30}	X_7	Distance covered at a running speed between $24\,km.h^{-1}$ and $30\,km.h^{-1}$
D_{30}	X_8	Distance covered at a running speed over $30\,km.h^{-1}$
HID_{15}	X_9	Distance covered at high intensity (over $30\,km.h^{-1}$)
HID_{21}	X_{10}	Distance covered at high intensity (over $21\,km.h^{-1}$)
Acc_3	X_{11}	Number of accelerations performed over $3\,m.s^{-2}$
$Acc_{3.5}$	X_{12}	Number of accelerations performed over $3.5\,m.s^{-2}$
Acc_4	X_{13}	Number of accelerations performed over $4\,m.s^{-2}$
Dec_3	X_{14}	Number of decelerations performed over $3\,m.s^{-2}$
$Dec_{3.5}$	X_{15}	Number of decelerations performed over $3.5\,m.s^{-2}$
Dec_4	X_{16}	Number of decelerations performed over $4\,m.s^{-2}$
$Sprint_{24}$	X_{17}	Number of running sprints over $24\,km.h^{-1}$
$Speed_{avg}$	X_{18}	Averaged running speed of the session ($km.h^{-1}$)
$Speed_{max}$	X_{19}	Maximal running speed reached during the session ($km.h^{-1}$)
Acc_{max}	X_{20}	Maximal accelerations performed during the session ($m.s^{-2}$)
Player	X_{22}	Player's id

A.2 Overview of the most selected features for predictive analysis tasks

Table A2. Top ten selected features for predictive tasks. A complete significance of feature extracted from raw data is provided by Christ et al.[1].

Feature	source
acc_3.5mss	commercial features
acc_3mss	commercial features
acc_4mss	commercial features
d_speed_0_5	commercial features
d_speed_10_15	commercial features
d_speed_15_21	commercial features
d_speed_5_10	commercial features
dec_3.5mss	commercial features
dec_3mss	commercial features
distance	commercial features
mean_second_derivative_central	raw data
partial_autocorrelation__lag_2	raw data
change_quantiles__f_agg_"var"__isabs_True__qh_0.8__ql_0.6	raw data
change_quantiles__f_agg_"mean"__isabs_False__qh_1.0__ql_0.6	raw data
change_quantiles__f_agg_"mean"__isabs_False__qh_1.0__ql_0.8	raw data
fft_coefficient__attr_"real"__coeff_75	raw data
fft_coefficient__attr_"imag"__coeff_90	raw data
friedrich_coefficients__coeff_2__m_3__r_30	raw data
friedrich_coefficients__coeff_3__m_3__r_30	raw data
matrix_profile__feature_"min"__threshold_0.98	raw data

A.3 Pseudo-code of the algorithms used in the methods

Algorithm 1: Algorithm of time-series forecasting models (auto-regression of target variable).

Input:

- **L**: Time window to predict the next sample from.
- **Y**: Time series for each player: $\{y_p^i\}_{(i \leq NGames, p \leq NPlayers)}$.
- **LearningAlgorithm**: Machine learning model used to learn the auto regressive task (RNN, Prophet, FOurTheta, FFT, Transformer).

$N \leftarrow |Y|$

$Y\_train, Y\_test \leftarrow Y[:0.8*N], [0.8*N:]$

$A \leftarrow LearningAlgorithm(Y\_train, input\_size = L, output\_size = 1, n\_epochs = 200)$

Output: MAPE(Y_test, A)

Algorithm 2: Algorithm of multivariate modeling with commercial features and features extracted from raw data - Individual Setting (I)

Input:

- **L**: Time window to consider past features to predict the target.
- **D**: dataset of each player: $\left\{ \left\{ \left(\left[X_p^i(t)\right]_{t \leq L}, y_p^i \right) \right\}_{i \leq NGames} \right\}_{p \leq NPlayers}$,

  where $\left[X_p^i(t)\right]_{t \leq L}$ is the p-th player's sequence of commercial features (see Section A.2) or learned Fourier features (see Methods) preceding the game of interest up to L days,

  and y_p^i is the actual performance of player p to predict for the game of interest.
- **LearningAlgorithm**: Machine learning model used to learn the regression (Ridge or LSTM)
- **PoolMethod**: method to aggregate each sequence $\left[X_p^i(t)\right]$ into a single vector representation X_p^i (mean or exp). `// As described in equations 1, 2.`
- **NFold**: number of folds use to perform cross validation evaluation.

$results \leftarrow []$

for $D_{player} \in D$ **do**

  $(X, y) \leftarrow D\_player$

  `/* Preprocessing */`

  $(X, y) \leftarrow MeanImputation(X, y)$

  $X \leftarrow MinMaxScaler(X, -1, 1)$

  $X \leftarrow VarianceThresholdVariableSelection(X, tresh = 0.1)$ `// as described in Methods`

  `/* End Preprocessing */`

  if $LearningAlgorithm \neq LSTM$ **then**

    $X \leftarrow PoolMethod(X)$ `// no pooling if LSTM model is used`

  $Splits \leftarrow RandomSplit(X, y, NFold)$

  $CV\_MAPE \leftarrow []$

  while $i < NFold$ **do**

    $X\_train, y\_train, X\_testy\_test = Splits[i]$

    $A \leftarrow LearningAlgorithm(X\_train, y\_train)$

    $CV\_MAPE[i] \leftarrow MAPE(X\_test, y\_test, A)$

  $results[player] \leftarrow mean(CV\_MAPE)$

Output: mean(results)

Algorithm 3: Algorithm of multivariate modeling with commercial features and features extracted from raw data - Group Setting (G)

Input:

- **L**: Time window to consider past features to predict the target.
- **D**: dataset of each player: $\left\{\left(\left[X_p^i(t)\right]_{t\leq L}, y_p^i\right)\right\}_{(i\leq NGames, p\leq NPlayers)}$
- **LearningAlgorithm**: Machine learning model used to learn the regression (Ridge or LSTM)
- **PoolMethod**: method to aggregate each sequence $\left[X_p^i(t)\right]$ into a single vector representation X_p^i (mean or exp). `// As described in Equations 1, 2`
- **NFold**: number of folds use to perform cross validation evaluation.

$(X,y) \leftarrow D$

`/* Preprocessing */`

$(X,y) \leftarrow MeanImputation(X,y)$

$X \leftarrow MinMaxScaler(X,-1,1)$

$X \leftarrow VarianceThresholdVariableSelection(X, tresh = 0.1)$ `// as described in Methods`

if $LearningAlgorithm \neq LSTM$ **then**

  $X \leftarrow PoolMethod(X)$ `// no pooling if LSTM model is used`

$Splits \leftarrow RandomSplit(X,y,NFold)$

$CV\_MAPE \leftarrow []$

while $i < NFold$ **do**

  $X\_train, y\_train, X\_testy\_test = Splits[i]$

  $A \leftarrow LearningAlgorithm(X\_train, y\_train)$

  $CV\_MAPE[i] = MAPE(X\_test, y\_test, A)$

Output: mean(CV_MAPE)

References

1. Christ, M., Braun, N. & Neuffer, J. Overview on time series feature extraction (tsfresh–a python package).
